# Allergens sensitization among children with allergic diseases in Shanghai, China: age and sex difference

**DOI:** 10.1186/s12931-022-02008-7

**Published:** 2022-04-15

**Authors:** Xiaolan Ying, Xinyi Qi, Yong Yin, Hongmei Wang, Hao Zhang, Haohua Jiang, Lin Yang, Jinhong Wu

**Affiliations:** 1 Department of Respiratory Medicine, Shanghai Children’s Medical Center, School of Medicine, Shanghai Jiao Tong University, No. 1678 Dongfang Road, Pudong, Shanghai, 200127 China; 2 Ya’an Polytechnic College Affiliated Hospital, No.132 Yucai Road, Sichuan 625000 Ya’an, China; 3 Shanghai Jiao Tong University School of Medicine, No. 227 South Chongqing Road, Huangpu, Shanghai, 200025 China; 4 Department of Clinical Laboratory, Shanghai Children’s Medical Center, School of Medicine, Shanghai Jiao Tong University, No. 1678 Dongfang Road, Pudong, Shanghai, 200127 China

**Keywords:** Allergic disease, Allergen, Aeroallergen, Children, Serum IgE, Sex difference, Age difference

## Abstract

**Background:**

The distribution of allergens has geographic characteristics. Local epidemiological data provides evidence-based strategies for the prevention and management of allergic diseases. Age and sex differences may exist in the prevalence of sensitivity to various allergens. We investigated the distribution of common allergens in allergic children in Shanghai, southeastern China.

**Methods:**

39,926 children 1 month to 18 years of age diagnosed with allergic diseases were tested for the presence of serum-specific Immunoglobulins E (sIgE) to 17 allergens common to this region, using a reversed enzyme allergosorbent test.

**Results:**

25,757 (64.5%) of the subjects showed elevated sIgE to at least one of the tested allergens. House mite and dust mite were the most common aeroallergens, while egg and milk were the most common food allergens. The most common aeroallergens and food allergens were similar among each allergic disease. By age-group analysis, the positive rates of aeroallergens were higher at older age. Several peaks of sensitization to food allergens were observed in children between 1 and 3 years of age for eggs, milk, nut, crab and shrimp. In addition, the sensitization to beef and mango was highest in children 3–6 years of age. The rate of positive sIgE detection was higher in males than females for all the tested allergens except cockroach, trees and beef. Considering the interplay between sex and ages and other related components (including season, monthly temperature, humidity, air quality index, test rate of patients), the sIgE positive rates of the main aeroallergens increased with age, while the main food allergens decreased; males are more sensitive to several aeroallergens (including dust mite, house mite, cat epithelium, dog epithelium and mulberry).

**Conclusions:**

House mite, dust mite, milk, and egg are major allergens in Shanghai. Children at younger age are more sensitive to food allergens, while increasing overall prevalence of sensitization can be found with increasing age. Boys have higher positive rates of sIgE responses than girls. Knowledge of the prevalence of allergen sensitization in different age groups and sex may help facilitate diagnosis and intervention efforts to mitigate the impact of allergic diseases in this large geographical region. This approach may be extrapolated to other regions.

**Supplementary Information:**

The online version contains supplementary material available at 10.1186/s12931-022-02008-7.

## Background

The growing prevalence and incidence of allergic diseases over recent years has caused increased concerns [1]. Complicated and relatively unclear mechanisms underlie the development of allergies. Therapy and prevention of allergic diseases largely rely on correct identification of the causative allergens and implementation of allergen avoidance.

The serum-specific Immunoglobulin E (sIgE) test, which is rapid and easy to perform, is widely used as the standard and reliable method for the identification of specific allergens [2].

The distribution of allergens varies according to the natural environment, e.g., climate, seasonal change, and lifestyle, e.g., food choices, presence of pets, in different geographical areas. In addition, susceptibility to various allergens may vary between different population subsets, for example, by age and sex [3, 4]. Several studies have analyzed the prevalence of aeroallergens and food allergens in China [5, 6]. However, few local large epidemiological studies about children with various allergic diseases have been conducted in Shanghai, a coastal area located in southeastern China with a population of 24 million people.

This study aimed to analyze the prevalence of common allergens and their distribution in children by age and sex in Shanghai, China. Understanding the epidemiology of allergen frequencies may provide evidence-based support for efforts to prevent and manage allergic diseases.

## Materials and methods

### Subjects

From December 2015 to September 2020, children 1 month to 18 years of age with clinically suspected allergic diseases, as evaluated by physicians at the Shanghai Children’s Medical Center affiliated to Shanghai Jiao Tong University School of Medicine, provided data for this study. A total of 39,926 patients with complete data were selected for further analysis.

### Methods

Ten common aeroallergens and seven food allergens were assessed for all patients. Aeroallergens included house mite (*Der p 1*), dust mite (*Der f 1*), cat epithelium, dog epithelium, cockroach, mold (mixture of Penicillium notatum, branch spore mildew, Aspergillus fumigates and Alternaria), trees (mixture of cypress, elm, phoenix tree, willow, and cottonwood), mulberry, grass (mixture of bluegrass, ryegrass, and timothy), amaranth. Food allergens included milk, beef, nut, egg, shrimp, crab, and mango.

Serum allergen-specific Immunoglobulin E (sIgE) levels of the patients were assayed by the AllergyScreen test (Mediwiss Analytic GmbH, Moers, Germany) with uniform and standardized procedures, according to the manufacturer’s manual. The minimum detection limit is 0.01 IU/mL. As per the method recommendation, results were divided into seven levels: level 0 (< 0.35 IU/mL), level 1 (0.35– < 0.70 IU/mL), level 2 (0.70– < 3.50 IU/mL), level 3 (3.5– < 17.5 IU/mL), level 4 (17.5– < 50 IU/mL), level 5 (50– < 100 IU/mL), level 6 (≥ 100 IU/mL). Although the standard procedure takes 0.35 IU/mL as the cut-off value to determine a positive sensitization, using this cut-off for sIgE testing has high sensitivity but poor specificity to diagnose allergy [7, 8]. For this study, sIgE levels ≥ 0.70 IU/mL (level 2 and higher) were considered to be positive. Results for the cut‐off value set as 0.35 IU/mL can also be seen in the Additional file 1.

### Data analysis

Data were classified and analyzed by using the IBM SPSS Statistics version 26. Percentage was used to describe categorical values, and chi-square test was used to test positive rates to different allergens stratified by age and sex. Statistical significance was based on *α* < 0.05. For multiple comparisons, *p* values below a Bonferroni-corrected *α* = 0.0025 (*α* = 0.05/20 outcomes) were also reported. Logistic regression model was performed to provide effect estimates for age, sex and the interplay between sex and ages. Season, weather (including monthly temperature, humidity, air quality index), test rate of patients (calculation: number of children tested divided by number of children entered into our hospital per month each year) were selected as the potential covariate in the model. Fifty-eight months were included in this research and monthly temperature, humidity, air quality index were defined as the average level per month. Stratified analysis was taken to understand the distribution of allergens in different allergic diseases.

## Results

### Demographic characteristics

A total of 39,926 patients (24,057 males, 15,869 females) were enrolled in this study during 2015–2020, with mean age of 5.02 ± 2.99 years (range: 1 month–18.0 years, interquartile range: 3.0–7.0 years). Children were divided into 5 age groups by developmental stages: 2373 infants (1 month–1 year), 5517 toddlers (> 1–3 years), 17,689 preschool children (> 3–6 years), 12,893 school aged children (> 6–12 years) and 1454 adolescents (> 12–18 years). The diagnoses included rhinitis (the most common disease, 17,336 cases), asthma, food allergy, conjunctivitis, eczema, urticaria and combining diagnoses of two or more allergic diseases (Table 1).

**Table 1:** Characteristics of participants

	Number of subjects	Percentage (%)
Age group		
1 month–1 years	2373	5.9
> 1–3 years	5517	13.8
> 3–6 years	17,689	44.3
> 6–12 years	12,893	32.3
> 12–18 years	1454	3.6
Sex		
Male	24,057	60.3
Female	15,869	39.7
Season		
Spring (March–May)	11,003	27.6
Summer (June–August)	13,052	32.7
Autumn (September–November)	9412	23.6
Winter (December–February)	6459	16.2
Diagnosis		
Rhinitis	17,336	43.4
Eczema/atopic dermatitis	8523	21.3
Urticaria	5348	13.4
Asthma	4193	10.5
Conjunctivitis	915	2.3
Food allergy	715	1.8
Multiple allergic disease^a^	2896	7.3

^a^A combination of 2 or more allergic diseases

### Allergen sensitization profiles

**Fig. 1:**
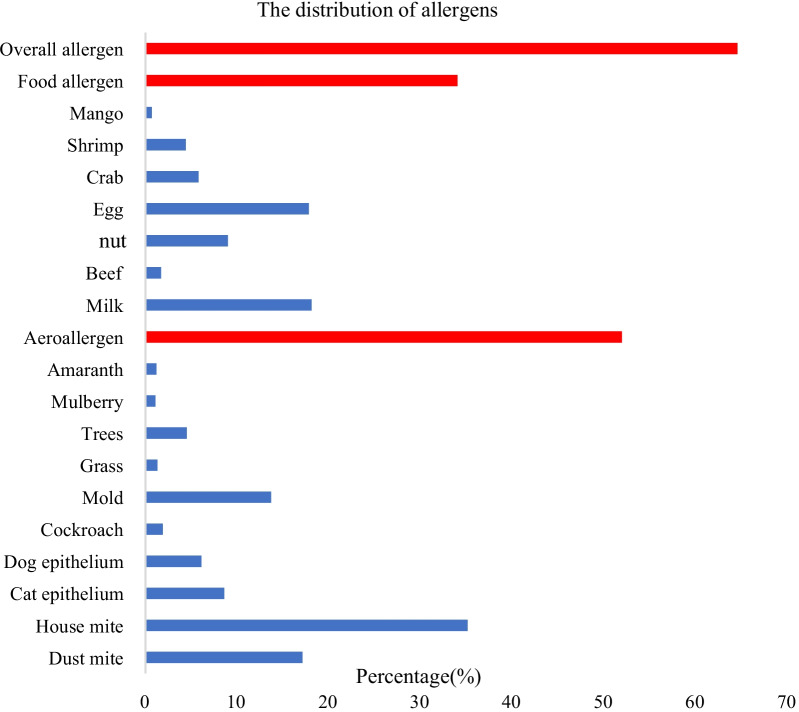
The distribution of allergens. Overall allergen: At least one positive allergen. Food allergen: At least one positive food allergen. Aeroallergen: At least one positive aeroallergen

The overall positive rate was 64.5% (25,757/39,926) (51.9% with at least one positive aeroallergen, 34.0% with at least one positive food allergen). House mite (the prevalence was 35.1%) and dust mite (17.1%) were the most common aeroallergen in Shanghai, followed by mold (13.7%), cat epithelium (8.6%) and dog epithelium (6.1%) (Fig. 1). The most frequently positive food allergen was milk (the prevalence was 18.1%), followed by egg (17.8%) and nut (9.0%). sIgE level of each allergen in different age groups or between males and females can be seen in Additional file 1: Table S1.

**Fig. 2:**
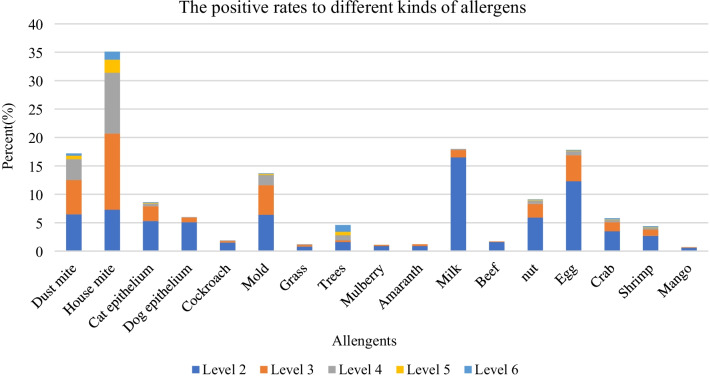
The positive rates to different kinds of allergens

Positive allergen results were more likely to be found at level 2–3 than at higher levels. House mite, dust mite, trees, mold, elicited a relatively high level of strong positive reaction (the positive rate of level 4–6 was 14.4%, 4.7%, 2.7%, 2.1%, respectively) (Fig. 2).

### Age difference of sensitization profiles (in allergens)

**Fig. 3:**
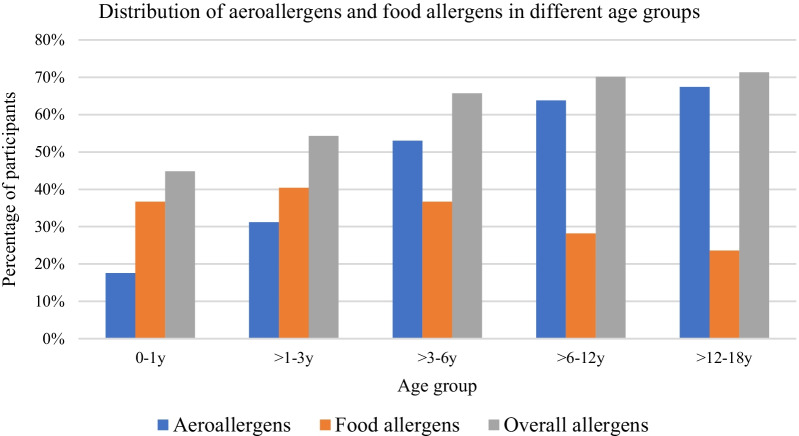
Distribution of aeroallergens and food allergens in different age groups

We found an association between age and the allergic positive rate. The overall positive rates and aeroallergen positive rates were higher in the older age groups. However, the positive rates of food allergens were highest in those aged 1–3 years (Fig. 3).

Significant statistical differences were observed in the positive rates of each specific aeroallergen (except amaranth) among the various age groups (Table 2). The positive rates to cockroach and trees were highest in those aged > 6–12 years and > 3–6 years, respectively. For other aeroallergens, children aged > 12–18 years had the highest positive proportions. The positive rates of house mite, dust mite, mold, grass and mulberry were increased with increasing age.

**Table 2:** Distribution of allergens in different age groups and sex

Allergens	Age group [n (%)]						*p*	Sex [n (%)]		*p*
	0–1 years (n = 2373)	> 1–3 years (n = 5517)	> 3–6 years (n = 17,689)	> 6–12 years (n = 12,893)	> 12–18 years (n = 1454)	Total (N = 39,926)		Male (n = 24,057)	Female (n = 15,869)	
Dust mite	34 (1.4)^a^	242 (4.4)^b^	2758 (15.6)^c^	3321 (25.8)^d^	461 (31.7)^e^	6816 (17.1)	** < 0.001**^#^	4391 (18.3)	2425 (15.3)	** < 0.001**^#^
House mite	55 (2.3)^a^	679 (12.3)^b^	6320 (35.7)^c^	6175 (47.9)^d^	778 (53.5)^e^	14,007 (35.1)	** < 0.001**^#^	8926 (37.1)	5081 (32.0)	** < 0.001**^#^
Cat epithelium	200 (8.4)^a,b,c^	536 (9.7)^c,d^	1369 (7.7)^b^	1149 (8.9)^a,c^	165 (11.3)^d^	3419 (8.6)	** < 0.001**^#^	2210 (9.2)	1209 (7.6)	** < 0.001**^#^
Dog epithelium	71 (3.0)^a^	303 (5.5)^b^	1144 (6.5)^b^	812 (6.3)^b^	102 (7.0)^b^	2432 (6.1)	** < 0.001**^#^	1596 (6.6)	836 (5.3)	**0.001**^#^
Cockroach	25 (1.1)^a^	77 (1.4)^a^	363 (2.1)^b^	279 (2.2)^b^	29 (2.0)^a,b^	773 (1.9)	** < 0.001**^#^	489 (2.0)	284 (1.8)	0.09
Mold	31 (1.3)^a^	273 (4.9)^b^	2357 (13.3)^c^	2502 (19.4)^d^	316 (21.7)^d^	5479 (13.7)	** < 0.001**^#^	3404 (14.1)	2075 (13.1)	**0.002**^#^
Grass	14 (0.6)^a^	42 (0.8)^a^	209 (1.2)^a^	206 (1.6)^b^	35 (2.4)^b^	506 (1.3)	** < 0.001**^#^	339 (1.4)	167 (1.1)	**0.002**^#^
Trees	70 (2.9)^a^	230 (4.2)^a,b^	874 (4.9)^b^	573 (4.4)^b^	64 (4.4)^a,b^	1811 (4.5)	** < 0.001**^#^	1119 (4.7)	692 (4.4)	0.17
Mulberry	10 (0.4)^a^	47 (0.9)^a,b^	202 (1.1)^b^	164 (1.3)^b^	24 (1.7)^b^	447 (1.1)	** < 0.001**^#^	304 (1.3)	143 (0.9)	**0.001**^#^
Amaranth	17 (0.7)^a^	69 (1.3)^a,b^	224 (1.3)^a,b^	148 (1.1)^a,b^	25 (1.7)^b^	483 (1.2)	0.06	320 (1.3)	163 (1.0)	**0.007**
Aeroallergen	417 (17.6)^a^	1719 (31.2)^b^	9375 (53.0)^c^	8230 (63.8)^d^	980 (67.4)^d^	20,721 (51.9)	** < 0.001**^#^	13,022 (54.1)	7699 (48.5)	** < 0.001**^#^
Milk	424 (17.9)^a,b^	1164 (21.1)^c^	3337 (18.9)^b^	2092 (16.2)^a,d^	206 (14.2)^d^	7223 (18.1)	** < 0.001**^#^	4541 (18.9)	2682 (16.9)	** < 0.001**^#^
Beef	7 (0.3)^a^	64 (1.2)^b^	344 (1.9)^c^	238 (1.8)^c^	22 (1.5)^b,c^	675 (1.7)	** < 0.001**^#^	419 (1.7)	256 (1.6)	0.33
Nut	157 (6.6)^a,b^	654 (11.9)^c^	1854 (10.5)^d^	870 (6.7)^b^	68 (4.7)^a^	3603 (9.0)	** < 0.001**^#^	2278 (9.5)	1325 (8.3)	** < 0.001**^#^
Egg	474 (20.0)^a^	1169 (21.2)^a^	3725 (21.1)^a^	1639 (12.7)^b^	106 (7.3)^c^	7113 (17.8)	** < 0.001**^#^	4211 (17.5)	2902 (18.3)	**0.045**
Crab	75 (3.2)^a^	488 (8.8)^b^	1252 (7.1)^c^	451 (3.5)^a^	44 (3.0)^a^	2310 (5.8)	** < 0.001**^#^	1492 (6.2)	818 (5.2)	** < 0.001**^#^
Shrimp	57 (2.4)^a^	348 (6.3)^b^	949 (5.4)^b^	369 (2.9)^a^	42 (2.9)^a^	1765 (4.4)	** < 0.001**^#^	1149 (4.8)	616 (3.9)	** < 0.001**^#^
Mango	22 (0.9)^a,b^	36 (0.7)^a,b^	151 (0.9)^b^	69 (0.5)^a^	10 (0.7)^a,b^	288 (0.7)	**0.015**	192 (0.8)	96 (0.6)	**0.026**
Food allergen	872 (36.7)^a^	2227 (40.4)^b^	6486 (36.7)^a^	3630 (28.2)^c^	343 (23.6)^d^	13,558 (34.0)	** < 0.001**^#^	8371 (34.8)	5187 (32.7)	** < 0.001**^#^
Overall allergen	1062 (44.8)^a^	2996 (54.3)^b^	11,626 (65.7)^c^	9037 (70.1)^d^	1036 (71.3)^d^	25,757 (64.5)	** < 0.001**^#^	15,937 (66.2)	9820 (61.9)	** < 0.001**^#^

Bolding indicates *p* < 0.05

^#^ indicates if below Bonferroni-adjusted *p* < 0.0025 (0.05/20 outcomes)

For the comparison of each allergen, significant differences between two age groups (*p* < 0.05)are indicated with different letters (a–e)

Among the food allergens tested, we also found that positive rates varied by age groups (Table 2). The positive rates to beef and mango were highest in those aged 3–6 years. Positive rates to other food allergens peaked in 1–3-year-olds. 12–18-year-olds had the lowest proportions for milk, nut, egg and crab positivity.

Table 3 summarizes the distribution of the highest levels of allergen sensitization (levels 4–6) for specific aeroallergens (dust mite, house mite, mold, trees) by age group. Positive rates increased with age.

**Table 3:** Distribution of high-level allergens in different age groups and sex

Allergens (level 4–6)	Age [n (%)]						*P*	Sex [n (%)]		*p*
	0–1 years (n = 2373)	> 1–3 years (n = 5517)	> 3–6 years (n = 17,689)	> 6–12 years (n = 12,893)	> 12–18 years (n = 1454)	Total (N = 39,926)		Male (n = 24,057)	Female (n = 15,869)	
Dust mite	2 (0.1)	15 (0.3)	561 (3.2)	1108 (8.6)	146 (10.0)	1832 (4.6)	** < 0.001**^#^	1188 (4.9)	644 (4.1)	** < 0.001**^#^
House mite	5 (0.2)	62 (1.1)	2327 (13.2)	3005 (23.3)	352 (24.2)	5751 (14.4)	** < 0.001**^#^	3683 (15.3)	2068 (13.0)	** < 0.001**^#^
Mold	0 (0.0)	9 (0.2)	245 (1.4)	523 (4.1)	73 (5.0)	850 (2.1)	** < 0.001**^#^	530 (2.2)	320 (2.0)	0.21
Trees	38 (1.6)	124 (2.2)	525 (3.0)	353 (2.7)	35 (2.4)	1075 (2.7)	** < 0.001**^#^	676 (2.8)	399 (2.5)	0.07

Bolding indicates *p* < 0.05

^#^Indicates if below Bonferroni-adjusted *p* < 0.0025 (0.05/20 outcomes)

### Sex difference of allergen profiles

**Fig. 4:**
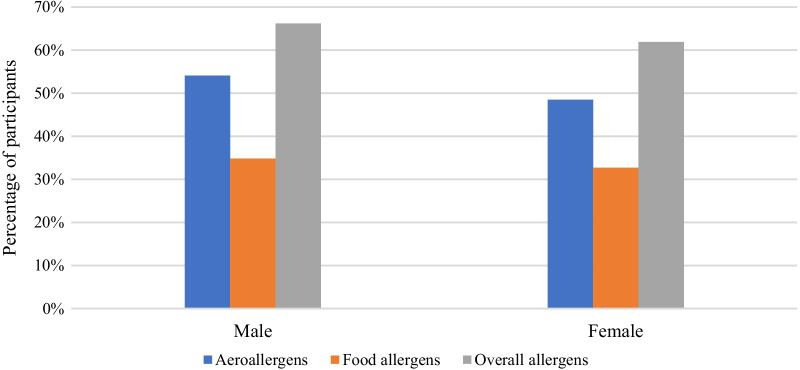
Distribution of aeroallergens and food allergens in males and females

Both aeroallergen and food allergens positive rate were overall higher in males than females (Fig. 4). However, no sex specific difference in the positive rates to allergens of cockroach, trees and beef was observed (Table 2).

In both males and females, house mite, dust mite, mold had the highest positive rates of aeroallergens while the highest positive percentage of food allergen were milk, egg, and nut (Table 2).

In addition to positivity rates, males had higher sIgE levels (levels 4–6) to dust and house mite allergens than females (Table 3).

We further explored the sex difference of allergen sensitization rate in various age groups. The sex specific positive rates to allergens in each age group were shown in Table 4. No significant differences in the positive rates to 4 allergens (cockroach, trees, mold, and beef) were observed between males and females in all age groups. For the remaining allergens that we tested, no significant differences in the positive rates of each allergen by sex (except mango) was found in the youngest group (0–1 year old). In all the other age groups, there were significant sex specific differences in the overall positive rate for both the aeroallergen and food allergen, though the sex-based differences varied by allergen and age. For example, males were more likely to be sIgE positive to cat/dog epithelium at ages 1–12 years, whereas sIgE levels in males against milk, crab and shrimp allergens were significantly higher than females in those aged 3–18 years (Table 4).

**Table 4:** Sex and age difference of positive rates (%)

Allergens	0–1 years (n = 2373)		*p*	> 1–3 years (n = 5517)		*p*	> 3–6 years (n = 17,689)		*p*	> 6–12 years (n = 12,893)		*p*	> 12-18y (n = 1454)		*p*
	Male (n = 1448)	Female (n = 925)		Male (n = 3216)	Female (n = 2301)		Male (n = 10,461)	Female (n = 7228)		Male (n = 8048)	Female (n = 4845)		Male (n = 884)	Female (n = 570)	
Dust mite	18 (1.2)	16 (1.7)	0.331	163 (5.1)	79 (3.4)	**0.003**	1723 (16.5)	1035 (14.3)	** < 0.001**^#^	2183 (27.1)	1138 (23.5)	** < 0.001**^#^	304 (34.4)	157 (27.5)	**0.006**
House mite	39 (2.7)	16 (1.7)	0.128	431 (13.4)	248 (10.8)	**0.003**	3922 (37.5)	2398 (33.2)	** < 0.001**^#^	4024 (50.0)	2151 (44.4)	** < 0.001**^#^	510 (57.7)	268 (47.0)	** < 0.001**^#^
Cat epithelium	130 (9.0)	70 (7.6)	0.228	338 (10.5)	198 (8.6)	**0.018**	881 (8.4)	488 (6.8)	** < 0.001**^#^	753 (9.4)	396 (8.2)	**0.022**	108 (12.2)	57 (10.0)	0.19
Dog epithelium	43 (3.0)	28 (3.0)	0.94	194 (6.0)	109 (4.7)	**0.037**	747 (7.1)	397 (5.5)	** < 0.001**^#^	543 (6.7)	269 (5.6)	**0.007**	69 (7.8)	33 (5.8)	0.14
Cockroach	13 (0.9)	12 (1.3)	0.35	48 (1.5)	29 (1.3)	0.47	227 (2.2)	136 (1.9)	0.18	186 (2.3)	93 (1.9)	0.14	15 (1.7)	14 (2.5)	0.31
Mold	17 (1.2)	14 (1.5)	0.48	169 (5.3)	104 (4.5)	0.21	1418 (13.6)	939 (13.0)	0.28	1596 (19.8)	906 (18.7)	0.12	204 (23.1)	112 (19.6)	0.12
Grass	9 (0.6)	5 (0.5)	0.80	22 (0.7)	20 (0.9)	0.44	140 (1.3)	69 (1.0)	**0.020**	146 (1.8)	60 (1.2)	**0.012**	22 (2.5)	13 (2.3)	0.80
Trees	41 (2.8)	29 (3.1)	0.67	146 (4.5)	84 (3.7)	0.10	521 (5.0)	353 (4.9)	0.77	376 (4.7)	197 (4.1)	0.11	35 (4.0)	29 (5.1)	0.31
Mulberry	7 (0.5)	3 (0.3)	0.56	35 (1.1)	12 (0.5)	**0.024**	139 (1.3)	63 (0.9)	**0.005**	110 (1.4)	54 (1.1)	0.22	13 (1.5)	11 (1.9)	0.50
Amaranth	11 (0.8)	6 (0.6)	0.75	45 (1.4)	24 (1.0)	0.24	144 (1.4)	80 (1.1)	0.12	100 (1.2)	48 (1.0)	0.19	20 (2.3)	5 (0.9)	**0.047**
Aeroallergen	261 (18)	156 (16.9)	0.47	1069 (33.2)	650 (28.2)	** < 0.001**^#^	5745 (54.9)	3630 (50.2)	** < 0.001**^#^	5319 (66.1)	2911 (60.1)	** < 0.001**^#^	628 (71.0)	352 (61.8)	** < 0.001**^#^
Milk	259 (17.9)	165 (17.8)	0.98	704 (21.9)	460 (20.0)	0.09	2067 (19.8)	1270 (17.6)	** < 0.001**^#^	1371 (17.0)	721 (14.9)	**0.001**^#^	140 (15.8)	66 (11.6)	**0.023**
Beef	4 (0.3)	3 (0.3)	0.83	36 (1.1)	28 (1.2)	0.74	206 (2.0)	138 (1.9)	0.78	158 (2.0)	80 (1.7)	0.20	15 (1.7)	7 (1.2)	0.48
Nut	99 (6.8)	58 (6.3)	0.59	394 (12.3)	260 (11.3)	0.28	1159 (11.1)	695 (9.6)	**0.002**^#^	583 (7.2)	287 (5.9)	**0.004**	43 (4.9)	25 (4.4)	0.67
Egg	288 (19.9)	186 (20.1)	0.90	716 (22.3)	453 (19.7)	**0.021**	2146 (20.5)	1579 (21.8)	**0.033**	996 (12.4)	643 (13.3)	0.14	65 (7.4)	41 (7.2)	0.91
Crab	41 (2.8)	34 (3.7)	0.25	297 (9.2)	191 (8.3)	0.23	791 (7.6)	461 (6.4)	**0.003**	327 (4.1)	124 (2.6)	** < 0.001**^#^	36 (4.1)	8 (1.4)	**0.004**
Shrimp	29 (2.0)	28 (3.0)	0.11	216 (6.7)	132 (5.7)	0.14	601 (5.7)	348 (4.8)	**0.007**	268 (3.3)	101 (2.1)	** < 0.001**^#^	35 (4.0)	7 (1.2)	**0.002**^#^
Mango	18 (1.2)	4 (0.4)	**0.044**	23 (0.7)	13 (0.6)	0.49	94 (0.9)	57 (0.8)	0.43	51 (0.6)	18 (0.4)	**0.048**	6 (0.7)	4 (0.7)	0.96
Food allergen	534 (36.9)	338 (36.5)	0.87	1339 (41.6)	888 (38.6)	**0.023**	3930 (37.6)	2556 (35.4)	**0.003**	2339 (29.1)	1291 (26.6)	**0.003**	229 (25.9)	114 (20.0)	**0.010**
Overall allergen	652 (45)	410 (44.3)	0.74	1806 (56.2)	1190 (51.7)	**0.001**^#^	7036 (67.3)	4590 (63.5)	** < 0.001**^#^	5790 (71.9)	3247 (67.0)	** < 0.001**^#^	653 (73.9)	383 (67.2)	**0.006**

Bolding indicates *p* < 0.05

^#^Indicates if below Bonferroni-adjusted *p* < 0.0025 (0.05/20 outcomes)

### Age or sex related to the positive rate of each allergen

This study showed that the allergen sensitivity had a prevalence pattern by age, considering the interplay between sex and ages and other components (including season, monthly temperature, humidity, air quality index, test rate of patients) (Table 5; Seasonal variation of allergens can be seen in Additional file 1: Fig. S5). The prevalence of the main aeroallergens (except cat epithelium, trees and amaranth) increased continuously with age, while for the main food allergens (except beef and mango), the sensitivity decreased. With 1-year increase, aeroallergen positive rate increased by 2–17% and food allergen positive rate decreased by 4–16%. In terms of the sex difference (Table 5), males are more sensitive to several aeroallergens (including dust mite, house mite, cat epithelium, dog epithelium and mulberry), which is in accordance with the univariate test. However, for food allergens, no sex difference was found by multiple analysis.

**Table 5:** Age or sex related to the positive rate of each allergen: logistic analysis

Allergens	Age [*p*, OR (95%CI)]	Sex [*p*, OR (95%CI)]	Age*Sex [*p*, OR (95%CI)]
Dust mite	** < 0.001**^#^, 1.17 (1.16–1.19)	**0.007**, 1.19 (1.05–1.35)	0.72, 1.00 (0.99–1.02)
House mite	** < 0.001**^#^, 1.14 (1.04–1.21)	**0.007**, 1.14 (1.04–1.24)	**0.045**, 1.02 (1.00–1.03)
Cat epithelium	0.06, 1.02 (1.00–1.04)	**0.002**^#^, 1.26 (1.09–1.45)	0.63, 0.99 (0.97–1.02)
Dog epithelium	**0.043**, 1.02 (1.00–1.05)	**0.005**, 1.28 (1.08–1.51)	0.97, 1.00 (0.97–1.03)
Cockroach	**0.028**, 1.05 (1.01–1.09)	0.36, 1.15 (0.85–1.56)	0.86, 1.00 (0.95–1.05)
Mold	** < 0.001**^#^, 1.14 (1.14–1.16)	0.35, 1.06 (0.94–1.20)	0.87, 1.00 (0.98–1.02)
Grass	** < 0.001**^#^, 1.10 (1.05–1.15)	0.24, 1.26 (0.86–1.84)	0.77, 1.01 (0.95–1.07)
Trees	0.07, 1.03 (1.00–1.05)	0.41, 1.09 (0.89–1.33)	0.84, 1.00 (0.96–1.03)
Mulberry	**0.018**, 1.06 (1.01–1.12)	**0.018**, 1.62 (1.09–2.42)	0.37, 0.97 (0.91–1.04)
Amaranth	0.78, 1.01 (0.96–1.06)	0.55, 1.12 (0.77–1.63)	0.38, 1.03 (0.97–1.10)
Aeroallergen	** < 0.001**^#^, 1.17 (1.16–1.19)	**0.005**, 1.13 (1.04–1.22)	**0.013**, 1.02 (1.01–1.04)
Milk	** < 0.001**^#^, 0.96 (0.95–0.98)	0.28, 1.06 (0.96–1.17)	**0.046**, 1.02 (1.00–1.04)
Beef	0.84, 1.00 (0.96–1.04)	0.67, 0.93 (0.69–1.28)	0.23, 1.03 (0.97–1.08)
Nut	** < 0.001**^#^, 0.94 (0.92–0.96)	0.16, 1.10 (0.96–1.27)	0.32, 1.01 (0.99–1.04)
Egg	** < 0.001**^#^, 0.91 (0.89–0.92)	0.47, 1.04 (0.94–1.15)	0.08, 0.98 (0.96–1.00)
Crab	** < 0.001**^#^, 0.84 (0.82–0.87)	0.18, 0.89 (0.76–1.06)	** < 0.001**^#^, 1.08 (1.05–1.12)
Shrimp	** < 0.001**^#^, 0.85 (0.82–0.88)	0.15, 0.87 (0.72–1.05)	** < 0.001**^#^, 1.09 (1.05–1.14)
Mango	0.08, 0.94 (0.87–1.01)	0.27, 1.31 (0.81–2.09)	0.92, 1.00 (0.92–1.10)
Food allergen	** < 0.001**^#^, 0.93 (0.92–0.94)	0.38, 1.04 (0.96–1.13)	**0.047**, 1.02 (1.00–1.03)
Overall allergen	** < 0.001**^#^, 1.08 (1.07–1.10)	**0.044**, 1.09 (1.00–1.18)	**0.008**, 1.02 (1.01–1.04)

Bolding indicates *p* < 0.05

^#^ indicates if below Bonferroni-adjusted *p* < 0.0025 (0.05/20 outcomes)

Adjusted by season, monthly temperature, humidity, air quality index, test rate of patients

### Age and sex difference of allergen sensitization in different diseases

The main aeroallergen and food allergen were similar in different allergic diseases (Table 6). The top 3 aeroallergens were house mite, dust mite and mold, respectively. Milk was the most common food allergen followed by egg and nut.

**Table 6:** Inter-disease distribution of allergen sensitization

Allergens	Asthma (n = 4193) (n, %)	Rhinitis (n = 17,336) (n, %)	Food allergy (n = 715) (n, %)	Conjunctivitis (n = 915) (n, %)	Eczema/atopic dermatitis (n = 8523) (n, %)	Urticaria (n = 5348) (n, %)	Multiple allergic disease (n = 2896) (n, %)
Dust mite	1022 (24.4)	3434 (19.8)	65 (9.1)	161 (17.6)	1049 (12.3)	355 (6.6)	730 (25.2)
House mite	2133 (50.9)	6562 (37.9)	114 (15.9)	340 (37.2)	2313 (27.1)	1028 (19.2)	1517 (52.4)
Cat epithelium	364 (8.7)	1399 (8.1)	47 (6.6)	75 (8.2)	827 (9.7)	407 (7.6)	300 (10.4)
Dog epithelium	245 (5.8)	1082 (6.2)	34 (4.8)	59 (6.4)	473 (5.5)	306 (5.7)	233 (8.0)
Cockroach	65 (1.6)	389 (2.2)	15 (2.1)	18 (2.0)	150 (1.8)	81 (1.5)	55 (1.9)
Mold	703 (16.8)	2662 (15.4)	57 (8.0)	132 (14.4)	912 (10.7)	515 (9.6)	498 (17.2)
Grass	38 (0.9)	231 (1.3)	9 (1.3)	9 (1.0)	129 (1.5)	30 (0.6)	60 (2.1)
Trees	150 (3.6)	827 (4.8)	32 (4.5)	30 (3.3)	443 (5.2)	193 (3.6)	136 (4.7)
Mulberry	44 (1.0)	156 (0.9)	7 (1.0)	7 (0.8)	148 (1.7)	40 (0.7)	45 (1.6)
Amaranth	56 (1.3)	187 (1.1)	18 (2.5)	9 (1.0)	130 (1.5)	42 (0.8)	41 (1.4)
Aeroallergen	2696 (64.3)	9635 (55.6)	236 (33.0)	484 (52.9)	3789 (44.5)	1939 (36.3)	1942 (67.1)
Milk	680 (16.2)	2967 (17.1)	158 (22.1)	186 (20.3)	1686 (19.8)	972 (18.2)	574 (19.8)
Beef	71 (1.7)	323 (1.9)	16 (2.2)	21 (2.3)	131 (1.5)	63 (1.2)	50 (1.7)
Nut	372 (8.9)	1271 (7.3)	99 (13.8)	60 (6.6)	1109 (13.0)	328 (6.1)	364 (12.6)
Egg	714 (17.0)	2779 (16.0)	180 (25.2)	162 (17.7)	1857 (21.8)	765 (14.3)	656 (22.7)
Crab	264 (6.3)	813 (4.7)	63 (8.8)	40 (4.4)	660 (7.7)	215 (4.0)	255 (8.8)
Shrimp	206 (4.9)	635 (3.7)	43 (6.0)	29 (3.2)	506 (5.9)	148 (2.8)	198 (6.8)
Mango	29 (0.7)	89 (0.5)	12 (1.7)	4 (0.4)	91 (1.1)	33 (0.6)	30 (1.0)
Food allergen	1380 (32.9)	5326 (30.7)	306 (42.8)	312 (34.1)	3426 (40.2)	1634 (30.6)	1174 (40.5)
Overall allergen	3054 (72.8)	11,345 (65.4)	397 (55.5)	599 (65.5)	5370 (63.0)	2764 (51.7)	2228 (76.9)

Children with multiple allergic disease had a higher positive rate of each aeroallergen compared with other diseases. The sIgE positive rates of food allergen were higher in children with food allergy.

Table 7 showed the relationship between age or sex and the positive rates of common allergens in patients with different allergic diseases. For children with asthma or rhinitis, the prevalence pattern by age can be seen in each allergen and results were similar with the whole participants. For other allergic diseases (except conjunctivitis), those three most common aeroallergens still had an increasing tendency. Food allergens decreased with age among children with eczema/atopic dermatitis and urticaria. Sex difference can only be found in rhinitis group.

**Table 7:** Relationship between age or sex and the positive rates of common allergens in different allergic diseases: logistic analysis

Allergens	Asthma		Rhinitis		Food allergy		Conjunctivitis		Eczema/atopic dermatitis		Urticaria	
	Age	Sex	Age	Sex	Age	Sex	Age	Sex	Age	Sex	Age	Sex
Dust mite												
* p*	** < 0.001**^#^	0.74	** < 0.001**^#^	**0.045**	** < 0.001**^#^	0.28	0.052	1.00	** < 0.001**^#^	0.36	** < 0.001**^#^	1.00
OR	1.17	0.94	1.16	1.23	1.38	1.67	1.11	1.00	1.20	1.12	1.15	1.00
House mite												
* p*	** < 0.001**^#^	0.06	** < 0.001**^#^	**0.007**	** < 0.001**^#^	0.79	0.13	0.97	** < 0.001**^#^	0.60	** < 0.001**^#^	0.72
OR	1.21	0.75	1.18	1.25	1.32	1.10	1.07	0.99	1.26	1.05	1.17	1.05
Mold												
* p*	** < 0.001**^#^	0.98	** < 0.001**^#^	0.41	**0.001**^#^	0.41	**0.013**	0.39	** < 0.001**^#^	0.92	** < 0.001**^#^	0.22
OR	1.16	1.00	1.10	1.09	1.29	1.44	1.15	1.55	1.21	0.99	1.10	0.81
Aeroallergen												
* p*	** < 0.001**^#^	0.34	** < 0.001**^#^	**0.001**^#^	** < 0.001**^#^	0.88	**0.027**	0.32	** < 0.001**^#^	0.87	** < 0.001**^#^	0.64
OR	1.27	0.86	1.16	1.30	1.35	1.04	1.10	1.43	1.21	1.01	1.10	1.05
Milk												
* p*	**0.003**	0.08	0.38	0.051	0.71	0.26	0.87	0.17	0.63	0.09	** < 0.001**^#^	0.59
OR	0.92	0.72	0.99	1.22	1.02	0.74	1.01	1.84	0.99	1.16	0.94	1.07
Nut												
* p*	**0.008**	0.20	0.11	0.06	0.24	0.31	0.19	0.72	0.10	0.30	0.30	0.18
OR	0.90	0.73	0.97	1.32	1.09	1.40	0.88	0.78	0.97	1.12	0.97	1.31
Egg												
* p*	** < 0.001**^#^	0.22	** < 0.001**^#^	0.58	0.38	0.84	0.10	0.79	** < 0.001**^#^	0.09	** < 0.001**^#^	0.40
OR	0.90	0.80	0.89	0.94	1.05	1.05	0.91	0.88	0.92	1.16	0.93	1.12
Food allergen												
* p*	** < 0.001**^#^	**0.048**	** < 0.001**^#^	0.14	0.49	0.83	0.28	0.47	** < 0.001**^#^	0.16	** < 0.001**^#^	0.13
OR	0.91	0.75	0.95	1.13	1.04	0.95	0.95	1.32	0.94	1.11	0.94	1.18
Overall allergen												
* p*	** < 0.001**^#^	0.06	** < 0.001**^#^	**0.019**	**0.004**	0.84	0.18	0.42	** < 0.001**^#^	0.25	**0.018**	0.26
OR	1.18	0.74	1.09	1.20	1.21	0.95	1.06	1.36	1.10	1.09	1.03	1.12

Bolding indicates *p* < 0.05

^#^Indicates if below Bonferroni-adjusted *p* < 0.0025 (0.05/20 outcomes)

Adjusted by season, monthly temperature, humidity, air quality index, test rate of patients, the interplay between age and sex

## Discussion

### Prevalence of immunologic evidence for allergen sensitization among children with allergic symptomatology

The prevalence of allergic diseases has increased greatly in China over the past 30 years. sIgE detection of specific allergens may facilitate the avoidance of the triggers and the implementation of allergen-specific immunotherapy.

64.5% of the children enrolled in the current study were identified as being sensitized to one or more allergens. Another study [9] reported an overall allergen sensitization of 69.1% in Guangzhou, a south region of China. However, since the tested allergens differed between studies, the overall positive rate could not be compared directly.

The positive rates of sensitization to specific inhaled allergens in this region ranged from 1.1% to 35.1%. House mite and dust mite were the most common, consistent with a previous study among adults in Shanghai [4]. Similarly, epidemiological studies in Qingdao [10] and Guangzhou [9] also found these two types to be major aeroallergens. Other studies found a higher prevalence of house mite than in the current one [11, 12]. Such discrepancies might be attributable to differences in subject recruitment. In the United States, the two most common allergens identified were Alternaria and Aspergillus [13].

The present results indicated that the percentage of food allergens with positivity ranged from 0.7% to 18.1%. Consistent with previous findings in the US and European countries, the most frequently positive food allergens were milk and egg [3, 9, 14–16]. Compared with other studies [3, 9, 14–16], sensitization to shrimp and crab was relatively lower.

Other than previous studies focused on patients with respiratory diagnosis such as asthma and rhinitis, a broad spectrum of patients with allergic symptoms were included in our study. The distribution of allergens was similar with the whole patients according to different diseases. Inhaled allergens had a relatively higher sensitization rate than the ingested allergens, which might suggest that the allergic diseases are more closely related to inhaled allergens in Shanghai. Climate may affect airborne and food allergens, as it determines the types of flora and fauna in certain geographical area [17]. Shanghai is a coastal city, located in southeastern China, characterized by high humidity suitable to the survival ofmites and mold.

Furthermore, the present study confirmed that the degree of allergen positivity was mainly in lower sIgE levels, indicating that the intensity of sensitization to these allergens was generally not extremely high. However, sIgE levels were not always parallel with the clinical severity of the disease [18]. It should be mentioned that a negative immunologic result does not mean the patients were not allergic. Since only the 17 common allergens were tested, other allergens may be responsible for the symptoms.

### Age-specific positive rates of different allergens

The present study assessed age specific features of allergens in different allergic diseases and revealed that the prevalence of inhalation and ingestion allergy varied by age. As the age increased, the prevalence of aeroallergens grew continuously; while a significant decrease in food sensitivity was detected (for those over 3 years). This was similar to the results of previous studies [9, 10, 14]. Compared with food allergens, inhaled allergens were the major contributors to diseases in patients aged > 3–18 years in our research.

As noted, the positive rate to inhaled allergens increased in children over 3 years old. This might be explained by an increase in exposure occurring with an increase in outdoor activity. Alternatively, it may reflect a maturing, more reactive immune system [5].

Food allergy diseases occur more frequently in younger individuals. Immature more permeable gastrointestinal mucosa, lesser gastrointestinal immune function, insufficient gastric acid secretion and lower levels of digestive enzymes are possible contributors [5]. In our study, milk and egg were the most common food allergens. The positive rates of egg and milk were the highest in the > 1–3 years group and decreased continuously with age. These results were similar to those of previous studies [19]. Milk is the earliest food given to babies, followed by eggs, and these are both commonly consumed foods by Chinese infants. Tolerance to foods is likely to be linked to a mature immune [20]. Therefore, sensitization to egg and milk primarily occurs in infants and young children.

This study indicates greater attention to prevent aeroallergen induced allergic diseases should be focused on older children, beyond toddler age. Improved ventilation and house cleaning are two ways to reduce exposure to aeroallergens. In contrast, since food allergens were more common in the youngest children, earlier efforts taken to detect developing disease might be helpful. It may be useful to check sIgE regularly to assess allergic status in order to identify allergens that are to be avoided or for desensitization therapies, especially for children with severe allergic symptoms.

### Sex-specific positive rates of different allergens

In the present study, the 5 most common allergens were the same for both boys and girls, namely milk, egg, house mite, dust mite, mold. However, the sex specific sIgE positive rates to allergens were different. Males were more susceptible to all 17 allergens, although statistical significance was not reached in each age group for each allergen. After adjusting the interplay between age and sex and other confounding factors, the sex difference was still statistically significant in several allergens. This result was consistent with the findings in previous studies [21, 22], even as the specific allergens tested in each study differed. In examining the subgroup of children with the higher sIgE levels, we also found the rate to be higher in boys than in girls. Moreover, other studies observed that boys had higher prevalence of allergic diseases compared with girls [22, 23]. It appears that boys may be more vulnerable to allergen sensitization and more susceptible to allergic diseases.

Why children exhibit these sex-based differences of positive allergen rates is still unclear. Possible reasons include lifestyle factors (opportunity of encountering different allergens), genetics, environment (including climate), immunological responses, sexual hormones, and cross-reactivity [23]. In addition, it is possible that symptom work-up bias accounts in part for this finding, such that fewer girls with symptoms get immunologic testing, but we have no data to support that speculation. Further studies are required to explain the mechanism of the sex-specific difference in allergen sensitization.

### Importance of allergen test for allergic diseases

Because clinical symptoms of allergic diseases are complex, some symptoms are obvious and some are not, allergen test aid in avoiding misdiagnosis. Excessive accumulation of seasonal and perennial allergens above the clinically relevant thresholds may give rise to allergy symptoms [24]. The detection of sIgE is conducive to identifying the status of allergy, i.e., the presence and degree of allergen response [2]. Identifying the specific allergens prevalent in a particular region may facilitate early diagnosis and inform development of strategies to prevent allergic diseases thereby helping to make appropriate preventive measures more effective. Since there are significant differences in patterns of sensitizations in patients from different age groups, age specific strategies of allergen exposure prevention should be taken.

While sIgE levels are generally associated with the severity of the disease, that is not always the case [18], especially after serious allergic reactions. In severe allergic reactions, the sIgE is consumed and false negative results may be obtained. We have also found (unpublished data) that sIgE levels can be elevated in some cases in children without evidence of clinical allergy related disease. At this point we don’t know whether such children are at increased risk of developing symptoms.

Although the diagnosis of allergic disease is confirmed by allergen test, the comprehensive analysis on the patients’ clinical symptoms and disease histories should never be neglected.

### Highlights and limitations

Our research has the following characteristics compared with previous studies: it examined a variety of common allergens (17), including food and aeroallergens simultaneously; we tested a large population with data collected over a 5-year period including various allergic diseases; we used an internationally recognized method of sIgE testing, ImmunoCAP, to detect sensitization; we categorized the severity of allergen sIgE response. Our findings may help clinicians and public health officials find effective interventions to specific groups of children and to direct researchers to conduct further studies on the epidemiology of allergic diseases. The present study provided evidence of the prevalence of allergies in our population and described sex and age specific differences to categories and specific allergens in children diagnosed with allergic diseases. Further comprehensive, in-depth and multi-center research is still required.

The present study has several limitations that need to be considered. First, only 17 allergens were assessed in the present study. The difference in tested allergen sources limits the comparability with other studies. And the duration between symptoms or allergen exposure and sIgE testing was not determined. It is possible that in some cases, testing occurred after declines in immunoglobulin levels leading to misclassifications as the serum half-life of IgE is 2–3 days [25]. In addition, only basic demographic and weather data were available in our study. We could not identify the effects of living area, lifestyle (diet, daily activities, the impact on sleep, et al.) on the patients’ changing features and their association with allergic symptoms, as these details are not included in their clinical records. Collecting more detailed information in the future through a standardized questionnaire may be helpful for further analysis. Also, the association with sIgE levels and the severity of allergic symptoms could not be analyzed as we didn’t have severity of illness scores to compare to the sIgE levels. Moreover, further studies are required to explore the pathogenic process of allergic diseases. Lastly, the results should be considered cautiously with the large number of comparisons as we mainly interpreted these based on raw estimates (some results did not survive Bonferroni-correction). However, adjustment for multiple comparisons might be too strict to find important findings that require further investigation [26]. Followed by this exploratory study, further researches were encouraged to provide a better understanding of the findings.

## Conclusions

In brief, the prevalence and distribution of the allergens among children with allergic diseases in Shanghai exhibited differences in terms of sex and age. Our approach, by defining the prevalence of specific allergens and risk groups for allergic diseases, may be extrapolatable to other regions. This in turn can guide mitigation efforts to reduce the burden of disease.

## Supplementary Information

**Additional file 1**: **Figure S1**. The distribution of allergens. **Figure S2**. The positive rates to different kinds of allergens. **Figure S3**. Distribution of aeroallergens and food allergens in different age groups. **Figure S4**. Distribution of aeroallergens and food allergens in males and females. **Figure S5**. Distribution of each aeroallergen and food allergen in different seasons. **Table S1**. The median and respective interquartile range of sIgE level. **Table S2.** Distribution of allergens in different age groups and sex. **Table S3**. Distribution of high-level allergens in different age groups and sex. **Table S4.** Sex and age difference of positive rates (%).**Table S5**. Age or sex related to the positive rate of each allergen: multiple logistic analysis. **Table S6**. Inter-disease distribution of allergen sensitization. **Table S7**. Relationship between age or sex and the positive rates of common allergens in different allergic diseases: multiple logistic analysis.

## Data Availability

The datasets analyzed during this present study are available from the corresponding author on reasonable request.

## Abbreviation

sIgE: Serum-specific immunoglobulins E
