# Supplementary material for: Allergens sensitization among children with allergic diseases in Shanghai, China: age and sex difference
Source: Respir Res. 2022 Apr 15;23:95. doi: 10.1186/s12931-022-02008-7 (PMC9013110; doi:10.1186/s12931-022-02008-7)
Supplement: Supplementary file 1 — Additional file 1: Figure S1. The distribution of allergens. Figure S2. The positive rates to different kinds of allergens. Figure S3. Distribution of aeroallergens and food allergens in different age groups. Figure S4. Distribution of aeroallergens and food allergens in males and females. Figure S5. Distribution of each aeroallergen and food allergen in different seasons. Table S1. The median and respective interquartile range of sIgE level. Table S2. Distribution of allergens in different age groups and sex. Table S3. Distribution of high-level allergens in different age groups and sex. Table S4. Sex and age difference of positive rates (%).Table S5. Age or sex related to the positive rate of each allergen: multiple logistic analysis. Table S6. Inter-disease distribution of allergen sensitization. Table S7. Relationship between age or sex and the positive rates of common allergens in different allergic diseases: multiple logistic analysis. [file 12931_2022_2008_MOESM1_ESM.docx]

Table1The mean level of sIgE (IU/mL) in different age group and sex

| Allergens | Age group (mean±SD) | | | | | |  | Sex (mean±SD) | | |
| --- | --- | --- | --- | --- | --- | --- | --- | --- | --- | --- |
|  | 0-1y | >1-3y | >3-6y | >6-12y | >12-18y | Total |  | Male | | Female |
| Dust mite | 0.08±1.21 | 0.24±2.46 | 1.76±7.41 | 4.38±13.11 | 5.48±15.13 | 2.43±9.58 |  | 2.59±9.76 | 2.20±9.31 | |
| House mite | 0.17±2.44 | 0.94±4.73 | 6.67±15.55 | 12.33±22.58 | 13.23±23.50 | 7.56±17.70 |  | 8.10±18.32 | 6.74±16.69 | |
| Cat epithelium | 0.24±1.18 | 0.44±2.67 | 0.52±3.79 | 0.76±5.14 | 1.10±6.54 | 0.59±4.19 |  | 0.67±4.61 | 0.46±3.46 | |
| Dog epithelium | 0.07±0.36 | 0.16±1.17 | 0.28±2.71 | 0.28±2.82 | 0.40±4.03 | 0.25±2.57 |  | 0.28±2.66 | 0.22±2.43 | |
| Cockroach | 0.04±0.63 | 0.12±2.23 | 0.15±2.24 | 0.08±1.04 | 0.07±0.44 | 0.11±1.81 |  | 0.12±1.86 | 0.10±1.74 | |
| Mold | 0.04±0.28 | 0.21±1.58 | 1.00±4.76 | 2.25±8.05 | 2.66±8.80 | 1.29±5.90 |  | 1.36±6.06 | 1.20±5.65 | |
| Grass | 0.02±0.12 | 0.03±0.36 | 0.08±1.47 | 0.14±1.93 | 0.37±4.91 | 0.10±1.75 |  | 0.12±2.00 | 0.07±1.29 | |
| Trees | 1.02±8.71 | 1.52±10.77 | 2.09±12.76 | 2.10±13.27 | 2.08±13.47 | 1.95±12.50 |  | 2.13±13.19 | 1.68±11.38 | |
| Mulberry | 0.02±1.00 | 0.02±0.23 | 0.06±0.98 | 0.06±0.68 | 0.08±0.86 | 0.05±0.78 |  | 0.06±0.91 | 0.04±0.51 | |
| Amaranth | 0.02±0.18 | 0.05±0.48 | 0.07±0.89 | 0.07±1.25 | 0.13±2.68 | 0.06±1.07 |  | 0.07±1.24 | 0.05±0.77 | |
| Milk | 0.55±3.25 | 0.52±2.53 | 0.51±2.59 | 0.49±2.57 | 0.61±3.80 | 0.51±2.67 |  | 0.53±2.62 | 0.48±2.75 | |
| Beef | 0.02±0.09 | 0.05±0.29 | 0.07±0.34 | 0.06±0.29 | 0.06±0.24 | 0.06±0.30 |  | 0.06±0.29 | 0.06±0.32 | |
| Nut | 0.51±3.54 | 0.75±4.51 | 0.64±4.33 | 0.53±4.29 | 0.69±6.55 | 0.61±4.40 |  | 0.68±4.76 | 0.52±3.79 | |
| Egg | 1.70±7.17 | 1.43±6.48 | 1.06±4.89 | 0.50±2.80 | 0.23±1.06 | 0.94±4.71 |  | 0.93±4.86 | 0.95±4.47 | |
| Crab | 0.50±4.75 | 0.83±6.10 | 0.62±5.49 | 0.46±5.31 | 0.77±7.56 | 0.60±5.57 |  | 0.70±6.22 | 0.45±4.40 | |
| Shrimp | 0.31±3.79 | 0.52±4.87 | 0.51±5.23 | 0.42±5.13 | 0.74±7.55 | 0.48±5.18 |  | 0.57±5.78 | 0.34±4.10 | |
| Mango | 0.06±1.32 | 0.03±0.32 | 0.04±0.89 | 0.04±1.14 | 0.03±0.15 | 0.04±0.94 |  | 0.04±0.61 | 0.04±1.29 | |

Table1The median and respective interquartile range of sIgE level

| Allergens | Age group [median (interquartile range)] | | | | | |  | Sex [median (interquartile range)] | | |
| --- | --- | --- | --- | --- | --- | --- | --- | --- | --- | --- |
|  | 0-1y  (n=2373) | >1-3y  (n=5517) | >3-6y  (n=17689) | >6-12y  (n=12893) | >12-18y  (n=1454) | Total  (N=39926) |  | Male  (n=24057) | | Female  (n=15869) |
| Dust mite | 0.00(0.00-0.00) | 0.00(0.00-0.00) | 0.06(0.00-0.06) | 0.00(0.00-0.77) | 0.00(0.00-1.60) | 0.00(0.00-0.13) |  | 0.00(0.00-0.19) | 0.00(0.00-0.02) | |
| House mite | 0.00(0.00-0.00) | 0.00(0.00-0.00) | 0.06(0.00-5.40) | 0.43(0.00-16.0) | 1.40(0.00-16.5) | 0.00(0.00-5.70) |  | 0.00(0.00-7.27) | 0.00(0.00-3.58) | |
| Mold | 0.00(0.00-0.00) | 0.10(0.00-0.00) | 0.06(0.00-0.00) | 0.00(0.00-0.23) | 0.00(0.00-0.40) | 0.00(0.00-0.00) |  | 0.00(0.00-0.00) | 0.00(0.00-0.00) | |
| Milk | 0.00(0.00-0.45) | 0.10(0.00-0.60) | 0.06(0.00-0.50) | 0.00(0.00-0.40) | 0.00(0.00-0.31) | 0.02(0.00-0.48) |  | 0.00(0.00-0.45) | 0.10(0.00-0.60) | |
| Nut | 0.00(0.00-0.00) | 0.00(0.00-0.09) | 0.00(0.00-0.04) | 0.00(0.00-0.00) | 0.00(0.00-0.00) | 0.00(0.00-0.00) |  | 0.00(0.00-0.02) | 0.00(0.00-0.00) | |
| Egg | 0.00(0.00-0.34) | 0.10(0.00-0.49) | 0.06(0.00-0.50) | 0.00(0.00-0.21) | 0.00(0.00-0.09) | 0.00(0.00-0.37) |  | 0.00(0.00-0.36) | 0.00(0.00-0.34) | |
| Others | 0.00(0.00-0.00) | 0.00(0.00-0.00) | 0.00(0.00-0.00) | 0.00(0.00-0.00) | 0.00(0.00-0.00) | 0.00(0.00-0.00) |  | 0.00(0.00-0.00) | 0.00(0.00-0.00) | |

Table 2 Distribution of allergens in different age group and sex

| Allergens | Age group[n (%)] | | | | | | *p* | Sex [n (%)] | | | *p* |
| --- | --- | --- | --- | --- | --- | --- | --- | --- | --- | --- | --- |
|  | 0-1y  (n=2373) | >1-3y  (n=5517) | >3-6y  (n=17689) | >6-12y  (n=12893) | >12-18y  (n=1454) | Total  (N=39926) |  | Male  (n=24057) | | Female  (n=15869) |  |
| Dust mite | 64(2.7) ^a^ | 379(6.9) ^b^ | 3491(19.7) ^c^ | 3900(30.2) ^d^ | 533(36.7) ^e^ | 8367(21.0) | **<0.001**^#^ | 5339(22.2) | 3028(19.1) | | **<0.001**^#^ |
| House mite | 104(4.4) ^a^ | 833(15.1) ^b^ | 6889(38.9) ^c^ | 6566(50.9) ^d^ | 830(57.1) ^e^ | 15222(38.1) | **<0.001**^#^ | 9649(40.1) | 5573(35.1) | | **<0.001**^#^ |
| Cat epithelium | 283(11.9) ^a,b,c^ | 729(13.2) ^c^ | 1813(10.2) ^b^ | 1452(11.3) ^a^ | 193(13.3) ^a,c^ | 4470(11.2) | **<0.001**^#^ | 2857(11.9) | 1613(10.2) | | **<0.001**^#^ |
| Dog epithelium | 132(5.6) ^a^ | 559(10.1) ^b^ | 1987(11.2) ^b^ | 1406(10.9) ^b^ | 173(11.9) ^b^ | 4257(10.7) | **<0.001**^#^ | 2740(11.4) | 1517(9.6) | | **<0.001**^#^ |
| Cockroach | 36(1.5) ^a^ | 150(2.7) ^b^ | 704(4.0) ^c^ | 548(4.3) ^c^ | 60(4.1) ^b,c^ | 1498(3.8) | **<0.001**^#^ | 920(3.8) | 578(3.6) | | 0.35 |
| Mold | 56(2.4) ^a^ | 405(7.3) ^b^ | 2969(16.8) ^c^ | 2949(22.9) ^d^ | 374(25.7) ^d^ | 6753(16.9) | **<0.001**^#^ | 4175(17.4) | 2578(16.2) | | **0.004** |
| Grass | 37(1.6) ^a^ | 100(1.8) ^a^ | 458(2.6) ^b^ | 372(2.9) ^b,c^ | 56(3.9) ^c^ | 1023(2.6) | **<0.001**^#^ | 658(2.7) | 365(2.3) | | **0.007** |
| Trees | 97(4.1) ^a^ | 319(5.8) ^b^ | 1221(6.9) ^c^ | 836(6.5) ^b,c^ | 100(6.9) ^b,c^ | 2573(6.4) | **<0.001**^#^ | 1569(6.5) | 1004(6.3) | | 0.44 |
| Mulberry | 36(1.5) ^a^ | 99(1.8) ^a^ | 408(2.3) ^a,b^ | 344(2.7) ^b,c^ | 52(3.6) ^c^ | 939(2.4) | **<0.001**^#^ | 609(2.5) | 330(2.1) | | **0.004** |
| Amaranth | 41(1.7) ^a^ | 138(2.5) ^a,b^ | 459(2.6) ^a,b^ | 293(2.3) ^a.b^ | 46(3.2) ^b^ | 977(2.4) | **0.021** | 613(2.5) | 364(2.3) | | 0.11 |
| Aeroallergen | 646(27.2) ^a^ | 2283(41.4) ^b^ | 10695(60.5) ^c^ | 8964(69.5) ^d^ | 1074(73.9) ^e^ | 23662(59.3) | **<0.001**^#^ | 14703(61.1) | 8959(56.5) | | **<0.001**^#^ |
| Milk | 705(29.7) ^a^ | 1896(34.4) ^b^ | 5601(31.7) ^a^ | 3408(26.4) ^c^ | 348(23.9) ^c^ | 11958(30.0) | **<0.001**^#^ | 7491(31.1) | 4467(28.1) | | **<0.001**^#^ |
| Beef | 36(1.5) ^a^ | 239(4.3) ^b^ | 1006(5.7) ^c^ | 748(5.8) ^c^ | 82(5.6) ^b,c^ | 2111(5.3) | **<0.001**^#^ | 1328(5.5) | 783(4.9) | | **0.010** |
| Nut | 232(9.8) ^a,b^ | 911(16.5) ^c^ | 2626(14.8) ^d^ | 1322(10.3) ^b^ | 113(7.8) ^a^ | 5204(13.0) | **<0.001**^#^ | 3262(13.6) | 1942(12.2) | | **<0.001**^#^ |
| Egg | 591(24.9) ^a^ | 1579(28.6) ^b^ | 5257(29.7) ^b^ | 2567(19.9) ^c^ | 209(14.4) ^d^ | 10203(25.6) | **<0.001**^#^ | 6095(25.3) | 4108(25.9) | | 0.22 |
| Crab | 112(4.7) ^a^ | 675(12.2) ^b^ | 1759(9.9) ^c^ | 728(5.6) ^a^ | 71(4.9) ^a^ | 3345(8.4) | **<0.001**^#^ | 2122(8.8) | 1223(7.7) | | **<0.001**^#^ |
| Shrimp | 86(3.6) ^a^ | 485(8.8) ^b^ | 1383(7.8) ^b^ | 562(4.4) ^a^ | 61(4.2) ^a^ | 2577(6.5) | **<0.001**^#^ | 1641(6.8) | 936(5.9) | | **<0.001**^#^ |
| Mango | 44(1.9) ^a^ | 88(1.6) ^a^ | 335(1.9) ^a^ | 228(1.8) ^a^ | 30(2.1) ^a^ | 725(1.8) | 0.59 | 458(1.9) | 267(1.7) | | 0.11 |
| Food allergen | 1208(50.9) ^a^ | 3061(55.5) ^b^ | 9317(52.7) ^a^ | 5553(43.1) ^c^ | 572(39.3) ^c^ | 19711(49.4) | **<0.001**^#^ | 12110(50.3) | 7601(47.9) | | **<0.001**^#^ |
| Overall allergen | 1412(59.5) ^a^ | 3754(68.0) ^b^ | 13505(76.3) ^c^ | 10075(78.1) ^d^ | 1167(80.3) ^d^ | 29913(74.9) | **<0.001**^#^ | 18326(76.2) | 11587(73.0) | | **<0.001**^#^ |

Bolding indicates *p* < 0.05.

# indicates if below Bonferroni-adjusted *p* < 0.0025 (0.05/20 outcomes).

For the comparison of each allergen, significant differences between two age groups  (*p* < 0.05) are indicated with different letters (a–e)

Table 3 Distribution of high-level allergens in different age group and sex

| Allergens (Level 4-6) | Age [n (%)] | | | | | | *p* | Sex [n (%)] | | | *p* |
| --- | --- | --- | --- | --- | --- | --- | --- | --- | --- | --- | --- |
|  | 0-1y  (n=2373) | >1-3y  (n=5517) | >3-6y  (n=17689) | >6-12y  (n=12893) | >12-18y  (n=1454) | Total  (N=39926) |  | Male  (n=24057) | | Female  (n=15869) |  |
| Dust mite | 2(0.1) | 15(0.3) | 561(3.2) | 1108(8.6) | 146(10.0) | 1832(4.6) | **<0.001**^#^ | 1188(4.9) | 644(4.1) | | **<0.001**^#^ |
| House mite | 5(0.2) | 62(1.1) | 2327(13.2) | 3005(23.3) | 352(24.2) | 5751(14.4) | **<0.001**^#^ | 3683(15.3) | 2068(13.0) | | **<0.001**^#^ |
| Mold | 0(0.0) | 9(0.2) | 245(1.4) | 523(4.1) | 73(5.0) | 850(2.1) | **<0.001**^#^ | 530(2.2) | 320(2.0) | | 0.21 |
| Trees | 38(1.6) | 124(2.2) | 525(3.0) | 353(2.7) | 35(2.4) | 1075(2.7) | **<0.001**^#^ | 676(2.8) | 399(2.5) | | 0.07 |

Bolding indicates *p* < 0.05.

# indicates if below Bonferroni-adjusted *p* < 0.0025 (0.05/20 outcomes).

Table 4 Sex and age difference of positive rates (%)

| Allergens | 0-1y(n=2373) | | *p* | >1-3y(n=5517) | | *p* | >3-6y(n=17689) | | | *p* | >6-12y(n=12893) | | *p* | >12-18y(n=1454) | | *p* |
| --- | --- | --- | --- | --- | --- | --- | --- | --- | --- | --- | --- | --- | --- | --- | --- | --- |
|  | Male  (n=1448) | Female  (n=925) |  | Male  (n=3216) | Female  (n=2301) |  | Male  (n=10461) | Female  (n=7228) | |  | Male  (n=8048) | Female  (n=4845) |  | Male  (n=884) | Female  (n=570) |  |
| Dust mite | 34(2.3) | 30(3.2) | 0.19 | 242(7.5) | 137(6.0) | **0.023** | 2170(20.7) | | 1321(18.3) | **<0.001**^#^ | 2546(31.6) | 1354(27.9) | **<0.001**^#^ | 347(39.3) | 186(32.6) | **0.011** |
| House mite | 64(4.4) | 40(4.3) | 0.91 | 515(16.0) | 318(13.8) | **0.025** | 4270(40.8) | 2619(36.2) | | **<0.001**^#^ | 4261(52.9) | 2305(47.6) | **<0.001**^#^ | 539(61.0) | 291(51.1) | **<0.001**^#^ |
| Cat epithelium | 181(12.5) | 102(11.0) | 0.28 | 463(14.4) | 266(11.6) | **0.002**^#^ | 1146(11.0) | 667(9.2) | | **<0.001**^#^ | 941(11.7) | 511(10.5) | **0.046** | 126(14.3) | 67(11.8) | 0.17 |
| Dog epithelium | 82(5.7) | 50(5.4) | 0.79 | 345(10.7) | 214(9.3) | 0.08 | 1264(12.1) | 723(10.0) | | **<0.001**^#^ | 932(11.6) | 474(9.8) | **0.002**^#^ | 117(13.2) | 56(9.8) | **0.050** |
| Cockroach | 18(1.2) | 18(1.9) | 0.17 | 95(3.0) | 55(2.4) | 0.20 | 411(3.9) | 293(4.1) | | 0.68 | 365(4.5) | 183(3.8) | 0.39 | 31(3.5) | 29(5.1) | 0.14 |
| Mold | 31(2.1) | 25(2.7) | 0.38 | 244(7.6) | 161(7.0) | 0.40 | 1787(17.1) | 1182(16.4) | | 0.20 | 1878(23.3) | 1071(22.1) | 0.11 | 235(26.6) | 139(24.4) | 0.35 |
| Grass | 21(1.5) | 16(1.7) | 0.59 | 53(1.6) | 47(2.0) | 0.28 | 286(2.7) | 172(2.4) | | 0.15 | 260(3.2) | 112(2.3) | **0.003** | 38(4.3) | 18(3.2) | 0.27 |
| Trees | 61(4.2) | 36(3.9) | 0.70 | 187(5.8) | 132(5.7) | 0.90 | 718(6.9) | 503(7.0) | | 0.80 | 547(6.8) | 289(6.0) | 0.06 | 56(6.3) | 44(7.7) | 0.31 |
| Mulberry | 27(1.9) | 9(1.0) | 0.08 | 67(2.1) | 32(1.4) | 0.06 | 258(2.5) | 150(2.1) | | 0.09 | 223(2.8) | 121(2.5) | 0.35 | 34(3.8) | 18(3.2) | 0.49 |
| Amaranth | 26(1.8) | 15(1.6) | 0.75 | 76(2.4) | 62(2.7) | 0.43 | 279(2.7) | 180(2.5) | | 0.47 | 199(2.5) | 94(1.9) | **0.049** | 33(3.7) | 13(2.3) | 0.12 |
| Aeroallergen | 399(27.6) | 247(26.7) | 0.65 | 1374(42.7) | 909(39.5) | **0.017** | 6504(62.2) | 4191(58.0) | | **<0.001**^#^ | 5742(71.3) | 3222(66.5) | **<0.001**^#^ | 684(77.4) | 390(68.4) | **<0.001**^#^ |
| Milk | 440(30.4) | 265(28.6) | 0.37 | 1141(35.5) | 755(32.8) | **0.040** | 3451(33.0) | 2150(29.7) | | **<0.001**^#^ | 2234(27.8) | 1174(24.2) | **<0.001**^#^ | 225(25.5) | 123(21.6) | 0.09 |
| Beef | 21(1.5) | 15(1.6) | 0.74 | 139(4.3) | 100(4.3) | 0.97 | 622(5.9) | 384(5.3) | | 0.07 | 498(6.2) | 250(5.2) | 0.16 | 48(5.4) | 34(6.0) | 0.67 |
| Nut | 139(9.6) | 93(10.1) | 0.71 | 543(16.9) | 368(16.0) | 0.38 | 1641(15.7) | 985(13.6) | | **<0.001**^#^ | 870(10.8) | 452(9.3) | **0.007** | 69(7.8) | 44(7.7) | 0.95 |
| Egg | 351(24.2) | 240(25.9) | 0.35 | 970(30.2) | 609(26.5) | **0.003** | 3078(29.4) | 2179(30.1) | | 0.30 | 1572(19.5) | 995(20.5) | 0.17 | 124(14.0) | 85(14.9) | 0.64 |
| Crab | 58(4.0) | 54(5.8) | **0.040** | 409(12.7) | 266(11.6) | 0.20 | 1104(10.6) | 655(9.1) | | **0.001**^#^ | 494(6.1) | 234(4.8) | **0.0****02**^#^ | 57(6.4) | 14(2.5) | **0.001**^#^ |
| Shrimp | 40(2.8) | 46(5.0) | **0.005** | 299(9.3) | 186(8.1) | 0.12 | 864(8.3) | 519(7.2) | | **0.009** | 389(4.8) | 173(3.6) | **0.001**^#^ | 49(5.5) | 12(2.1) | **0.001**^#^ |
| Mango | 32(2.2) | 12(1.3) | 0.11 | 54(1.7) | 34(1.5) | 0.56 | 201(1.9) | 134(1.9) | | 0.75 | 153(1.9) | 75(1.5) | 0.14 | 18(2.0) | 12(2.1) | 0.93 |
| Food allergen | 739(51.0) | 469(50.7) | 0.87 | 1827(56.8) | 1234(53.6) | **0.019** | 5646(54.0) | 3671(50.8) | | **<0.001**^#^ | 3544(44.0) | 2009(41.5) | **0.004** | 354(40.0) | 218(38.2) | 0.49 |
| Overall allergen | 855(59.0) | 557(60.2) | 0.57 | 2228(69.3) | 1526(66.3) | **0.020** | 8125(77.7) | 5380(74.4) | | **<0.001**^#^ | 6393(79.4) | 3682(76.0) | **<0.001**^#^ | 725(82.0) | 442(77.5) | **0.037** |

Bolding indicates *p* < 0.05.

# indicates if below Bonferroni-adjusted *p* < 0.0025 (0.05/20 outcomes).

Table 5 Age or sex related to the positive rate of each allergen: multiple logistic analysis

| Allergens | Age  [*p*, OR (95%CI)] | Sex  [*p*, OR (95%CI)] | Age*Sex  [*p*, OR (95%CI)] |
| --- | --- | --- | --- |
| Dust mite | **<0.001**^#^,  1.16(1.15-1.18) | **0.021**,  1.14(1.02-1.28) | 0.43,  1.01(0.99-1.02) |
| House mite | **<0.001**^#^,  1.19(1.18-1.21) | **0.01****3**,  1.12(1.03-1.22) | **0.041**,  1.02(1.00-1.03) |
| Cat epithelium | 0.94,  1.00(0.98-1.02) | **0.003**,  1.21(1.07-1.38) | 0.71,  1.00(0.98-1.02) |
| Dog epithelium | 0.13,  1.01(1.00-1.03) | **0.029**,  1.16(1.02-1.32) | 0.39,  1.01(0.99-1.03) |
| Cockroach | **<0.001**^#^,  1.05(1.02-1.08) | 0.71,  1.04(0.84-1.30) | 0.92,  1.00(0.96-1.053 |
| Mold | **<0.001**^#^,  1.14(1.14-1.16) | 0.27,  1.06(0.95-1.19) | 0.91,  1.00(0.98-1.02) |
| Grass | 0.27,  1.02(0.99-1.06) | 0.77,  0.96(0.74-1.25) | 0.09,  1.04(0.99-1.08) |
| Trees | 0.17,  1.02(0.99-1.04) | 0.94,  0.99(0.84-1.17) | 0.66,  1.01(0.98-1.04) |
| Mulberry | **0.013**,  1.05(1.01-1.08) | 0.09,  1.27(0.97-1.67) | 0.65,  0.99(0.95-1.03) |
| Amaranth | 0.26,  0.98(0.94-1.02) | 0.39,  0.89(0.69-1.16) | 0.06,  1.04(1.00-1.09) |
| Aeroallergen | **<0.001**^#^,  1.15(1.14-1.17) | 0.10  1.07(0.99-1.17) | **<0.001**^#^,  1.02(1.01-1.04) |
| Milk | **<0.001**^#^,  0.96(0.95-0.97) | **0.030,**  1.10(1.01-1.20) | 0.10,  1.01(1.00-1.03) |
| Beef | 0.43,  1.01(0.99-1.03) | 0.39,  1.08(0.90-1.30) | 0.73,  1.01(0.98-1.04) |
| Nut | **<0.001**^#^,  0.95(0.93-0.97) | 0.09,  1.11(0.98-1.24) | 0.50,  1.01(0.99-1.03) |
| Egg | **<0.001**^#^,  0.93(0.91-0.94) | 0.16,  1.07(0.97-1.17) | **0.037**,  0.98(0.97-1.00) |
| Crab | **<0.001**^#^,  0.87(0.85-0.89) | 0.34,  0.93(0.81-1.08) | **<0.001**^#^,  1.06(1.03-1.09) |
| Shrimp | **<0.001**^#^,  0.87(0.84-0.89) | 0.13,  0.88(0.75-1.04) | **<0.001**^#^,  1.07(1.04-1.10) |
| Mango | 0.53,  0.99(0.95-1.03) | 0.23,  1.20(0.89-1.62) | 0.65,  0.99(0.94-1.04) |
| Food allergen | **<0.001**^#^,  0.94(0.93-0.95) | **0.013**  1.11(1.02-1.20) | 0.72,  1.00(0.99-1.02) |
| Overall allergen | **<0.001**^#^,  1.06(1.05-1.08) | 0.12,  1.07(0.98-1.18) | **0.027**,  1.02(1.00-1.04) |

Bolding indicates *p* < 0.05.

# indicates if below Bonferroni-adjusted *p* < 0.0025 (0.05/20 outcomes).

Adjusted by season, monthly temperature, humidity, air quality index, test rate of patients

Table 6 Inter-disease distribution of allergen sensitization

| Allergens | Asthma (n=4193) (n,%) | Rhinitis (n=17336) (n,%) | Food allergy(n=715) (n,%) | Conjunctivitis (n=915) (n,%) | Eczema/atopic dermatitis (n=8523) (n,%) | Urticaria (n=5348) (n,%) | Multiple allergic disease(n=2896) (n,%) |
| --- | --- | --- | --- | --- | --- | --- | --- |
| Dust mite | 1221(29.1) | 4180(24.1) | 87(12.2) | 199(21.7) | 1302(15.3) | 499(9.3) | 879(30.4) |
| House mite | 2232(53.2) | 7127(41.1) | 139(19.4) | 374(40.9) | 2542(29.8) | 1218(22.8) | 1590(54.9) |
| Cat epithelium | 481(11.5) | 1844(10.6) | 63(8.8) | 96(10.5) | 1043(12.2) | 553(10.3) | 390(13.5) |
| Dog epithelium | 442(10.5) | 1895(10.9) | 72(10.1) | 115(12.6) | 809(9.5) | 546(10.2) | 378(13.1) |
| Cockroach | 132(3.1) | 728(4.2) | 25(3.5) | 39(4.3) | 283(3.3) | 161(3.0) | 130(4.5) |
| Mold | 852(20.3) | 3265(18.8) | 75(10.5) | 167(18.3) | 1155(13.6) | 652(12.2) | 587(20.3) |
| Grass | 103(2.5) | 449(2.6) | 11(1.5) | 24(2.6) | 255(3.0) | 88(1.6) | 93(3.2) |
| Trees | 235(5.6) | 1148(6.6) | 41(5.7) | 46(5.0) | 598(7.0) | 295(5.5) | 210(7.3) |
| Mulberry | 105(2.5) | 367(2.1) | 15(2.1) | 16(1.7) | 247(2.9) | 97(1.8) | 92(3.2) |
| Amaranth | 103(2.5) | 412(2.4) | 24(3.4) | 16(1.7) | 250(2.9) | 94(1.8) | 78(2.7) |
| Aeroallergen | 2947(70.3) | 10858(62.6) | 298(41.7) | 558(61.0) | 4447(52.2) | 2453(45.9) | 2101(72.5) |
| Milk | 1129(26.9) | 4932(28.4) | 243(34.0) | 321(35.1) | 2754(32.3) | 1651(30.9) | 928(32.0) |
| Beef | 218(5.2) | 997(5.8) | 35(4.9) | 56(6.1) | 378(4.4) | 245(4.6) | 182(6.3) |
| Nut | 545(13.0) | 1923(11.1) | 126(17.6) | 106(11.6) | 1477(17.3) | 526(9.8) | 501(17.3) |
| Egg | 1041(24.8) | 4143(23.9) | 225(31.5) | 241(26.3) | 2515(29.5) | 1137(21.3) | 901(31.1) |
| Crab | 381(9.1) | 1220(7.0) | 93(13.0) | 55(6.0) | 915(10.7) | 339(6.3) | 342(11.8) |
| Shrimp | 301(7.2) | 969(5.6) | 72(10.1) | 43(4.7) | 686(8.0) | 230(4.3) | 276(9.5) |
| Mango | 79(1.9) | 281(1.6) | 22(3.1) | 15(1.6) | 180(2.1) | 86(1.6) | 62(2.1) |
| Food allergen | 1993(47.5) | 8051(46.4) | 414(57.9) | 480(52.5) | 4682(54.9) | 2491(46.6) | 1600(55.2) |
| Overall allergen | 3388(80.8) | 13076(75.4) | 489(68.4) | 699(76.4) | 6302(73.9) | 3522(65.9) | 2437(84.2) |

Table 7 Relationship between age or sex and the positive rates of common allergens in different allergic diseases: multiple logistic analysis

| Allergens | Asthma | |  | Rhinitis | |  | Food allergy | | |  | Conjunctivitis | |  | Eczema/atopic dermatitis | |  | Urticaria | |  |
| --- | --- | --- | --- | --- | --- | --- | --- | --- | --- | --- | --- | --- | --- | --- | --- | --- | --- | --- | --- |
|  | Age | Sex |  | Age | Sex |  | Age | Sex |  | | Age | Sex |  | Age | Sex |  | Age | Sex |  |
| Dust mite | | | | | | | | | | | | | | | | | | | |
| *p* | **<0.001**^#^ | 0.25 |  | **<0.001**^#^ | 0.13 |  | **<0.001**^#^ | 0.82 |  | | **0.002**^#^ | 0.41 |  | **<0.001**^#^ | 0.26 |  | **<0.001**^#^ | 0.90 |  |
| OR | 1.14 | 0.82 |  | 1.14 | 1.15 |  | 1.30 | 1.09 |  | | 1.18 | 1.48 |  | 1.20 | 1.34 |  | 1.14 | 0.98 |  |
| House mite | | | | | | | | | | | | | | | | | | | |
| *p* | **<0.001**^#^ | 0.08 |  | **<0.001**^#^ | **0.021** |  | **<0.001**^#^ | 0.74 |  | | 0.09 | 0.70 |  | **<0.001**^#^ | 0.70 |  | **<0.001**^#^ | 0.94 |  |
| OR | 1.21 | 0.76 |  | 1.16 | 1.20 |  | 1.31 | 0.89 |  | | 1.08 | 1.15 |  | 1.25 | 1.04 |  | 1.16 | 1.01 |  |
| Mold | | | | | | | | | | | | | | | | | | | |
| *p* | **<0.001**^#^ | 0.45 |  | **<0.001**^#^ | 0.42 |  | **0.001**^#^ | 0.28 |  | | **0.012** | 0.27 |  | **<0.001**^#^ | 0.74 |  | **<0.001**^#^ | 0.06 |  |
| OR | 1.16 | 1.14 |  | 1.09 | 1.08 |  | 1.26 | 1.52 |  | | 1.14 | 1.68 |  | 1.20 | 1.04 |  | 1.07 | 0.74 |  |
| Aeroallergen | | | | | | | | | | | | | | | | | | | |
| *p* | **<0.001**^#^ | 0.56 |  | **<0.001**^#^ | **0.032** |  | **<0.001**^#^ | 0.80 |  | | **0.023** | 0.16 |  | **<0.001**^#^ | 0.84 |  | **<0.001**^#^ | 0.56 |  |
| OR | 1.27 | 0.91 |  | 1.13 | 1.19 |  | 1.30 | 1.07 |  | | 1.11 | 1.68 |  | 1.19 | 1.02 |  | 1.07 | 0.94 |  |
| Milk | | | | | | | | | | | | | | | | | | | |
| *p* | **<0.001**^#^ | 0.10 |  | 0.06 | **0.001**^#^ |  | 0.76 | 0.18 |  | | 0.57 | 0.11 |  | 0.22 | **0.029** |  | **<0.001**^#^ | 0.48 |  |
| OR | 0.91 | 0.77 |  | 0.98 | 1.32 |  | 0.98 | 0.73 |  | | 0.97 | 1.85 |  | 0.99 | 1.18 |  | 0.93 | 1.08 |  |
| Nut | | | | | | | | | | | | | | | | | | | |
| *p* | **<0.001**^#^ | 0.09 |  | 0.15 | 0.054 |  | 0.38 | 0.37 |  | | **0.01****0** | 0.13 |  | 0.11 | 0.37 |  | 0.73 | **0.027** |  |
| OR | 0.89 | 0.70 |  | 0.98 | 1.26 |  | 1.06 | 1.30 |  | | 0.79 | 0.41 |  | 0.98 | 1.09 |  | 1.00 | 1.44 |  |
| Egg | | | | | | | | | | | | | | | | | | | |
| *p* | **0.001**^#^ | 0.63 |  | **<0.001**^#^ | 0.71 |  | 0.59 | 0.67 |  | | 0.11 | 0.91 |  | **<0.001**^#^ | 0.03^#^ |  | **<0.001**^#^ | 0.55 |  |
| OR | 0.93 | 0.93 |  | 0.90 | 0.97 |  | 1.03 | 1.11 |  | | 0.92 | 0.95 |  | 0.95 | 1.18 |  | 0.95 | 1.07 |  |
| Food allergen | | | | | | | | | | | | | | | | | | | |
| *p* | **<0.001**^#^ | 0.08 |  | **<0.001**^#^ | 0.05 |  | 0.71 | 0.23 |  | | 0.35 | 0.27 |  | **<0.001**^#^ | **0.009** |  | **<0.001**^#^ | 0.07 |  |
| OR | 0.90 | 0.79 |  | 0.94 | 1.16 |  | 0.98 | 0.76 |  | | 0.96 | 1.49 |  | 0.96 | 1.21 |  | 0.95 | 1.20 |  |
| Overall allergen | | | | | | | | | | | | | | | | | | | |
| *p* | **<0.001**^#^ | **0.039** |  | **<0.001**^#^ | 0.06 |  | 0.07 | 0.28 |  | | 0.17 | 0.46 |  | **<0.001**^#^ | 0.15 |  | 0.40 | 0.61 |  |
| OR | 1.14 | 0.69 |  | 1.07 | 1.17 |  | 1.13 | 0.76 |  | | 1.07 | 1.36 |  | 1.09 | 1.12 |  | 1.01 | 1.05 |  |

Bolding indicates *p* < 0.05.

# indicates if below Bonferroni-adjusted *p* < 0.0025 (0.05/20 outcomes).

Adjusted by season, monthly temperature, humidity, air quality index, test rate of patients, the interplay between age and sex

sIgE levels≥0.35 IU/mL (level 1 and higher) were considered to be positive in the tables above.

Figure 1 The distribution of allergens

Overall allergen: At least one positive allergen

Food allergen: At least one positive food allergen

Aeroallergen: At least one positive aeroallergen

Figure 2 The positive rates to different kinds of allergens

Figure 3 Distribution of aeroallergens and food allergens in different age groups

Figure 4 Distribution of aeroallergens and food allergens in males and females

Figure 5 Distribution of each aeroallergen and food allergen in different seasons
